# An Unusual Case of Extranodal Diffuse Large B-Cell Lymphoma Infiltrating Skeletal Muscle: A Case Report and Review of the Literature

**DOI:** 10.1155/2016/9104839

**Published:** 2016-05-10

**Authors:** Joseph Hatem, Agata M. Bogusz

**Affiliations:** Department of Pathology and Laboratory Medicine, the University of Pennsylvania, Philadelphia, PA 19104, USA

## Abstract

Diffuse large B-cell lymphoma is extranodal in approximately 40% of cases, arising in nearly any organ system. DLBCL involvement of soft tissue and in particular skeletal muscle is extremely rare, comprising less than 1% of all extranodal non-Hodgkin lymphomas (NHL). We report a case of a 79-year-old man that presented with a DLBCL of the left triceps. In particular, we describe an unusual histologic appearance of pseudoglandular structures, resembling adenocarcinoma. We performed a review of lymphoma cases involving skeletal muscle diagnosed at our institution over the past 15 years as well as thorough PubMed review of the literature. We discuss the features of lymphoma involving skeletal muscle as it pertains to clinical characteristics, histologic subtype, tumor localization, diagnostic studies, therapy, and outcome. Finally, we highlight the diagnostic difficulties that can present in these rare and often challenging cases.

## 1. Introduction

Diffuse large B-cell lymphoma (DLBCL) is the most common non-Hodgkin lymphoma (NHL), accounting for approximately 30–40% of cases [1, 2]. DLBCL is heterogeneous in its clinical presentation, response to treatment, and biology [3]. The current standard of care is R-CHOP (rituximab, cyclophosphamide, doxorubicin, vincristine, and prednisone), which results in cure rates exceeding 50% [3]. An accurate diagnosis and the International Prognostic Index (IPI), a clinical tool developed by oncologists, which takes into account the patient's age, stage of disease, lactate dehydrogenase (LDH) level, performance status, and number of extranodal sites (one or more than one), are together the most important factors in determining therapy modalities and predicting outcome of patients with aggressive NHL [3].

DLBCL typically arises in lymph nodes; however, initial extranodal sites are identified in about 30–40% of cases. Any organ system may be affected, although involvement of the gastrointestinal system is most common [1]. Primary skeletal muscle involvement by DLBCL is exceedingly rare, comprising less than 1% of cases, with the majority of cases reported arising in the lower extremity, particularly thigh and calf region [4–7]. A variety of morphologic variants have been described [8, 9]. We describe a case of primary muscle DLBCL of the upper extremity with unusual histologic features. We performed a review of lymphoma cases involving skeletal muscle diagnosed at our institution over the last past 15 years, as well as thorough PubMed review of the literature. Finally, we discuss the diagnostic challenges and unique clinicopathologic characteristics of lymphomas involving skeletal muscle.

## 2. Case Report

**Figure 1:**
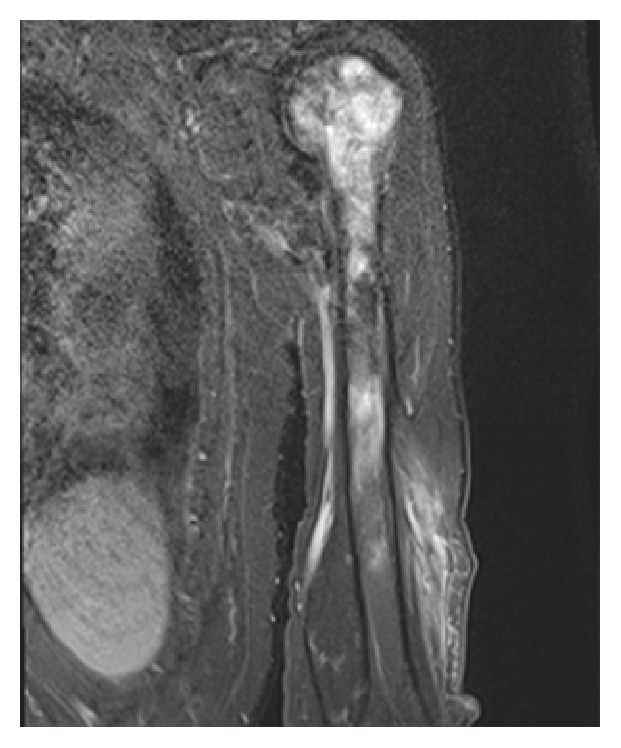
MRI of the left shoulder demonstrates a soft tissue lesion adjacent to the proximal humerus and potential bony lesions.

A 72-year-old man presented to his primary care physician (PCP) with neck pain of three-day duration, described as radiating to the upper back and shoulders. He had recalled lifting heavy wood and working a snow blower before the onset of symptoms. His past medical history is significant for hypertension, atherosclerotic disease with previous myocardial infarction (status postcoronary bypass ×3), hypercholesterolemia, hypothyroidism, and gastroesophageal reflux disease. On physical exam, neck stiffness with paraspinal tenderness in the right posterior neck was noted, aggravated by passive and active range of motion of the left shoulder. No neurologic signs such as tingling or numbness to the extremities, arm weakness, or gait aberrancies were noted. No cervical lymphadenopathy was present. A cervical spine X-ray revealed mild degenerative changes including endplate sclerosis, hypertrophic spurring, and disc space narrowing without erosions or unusual calcifications. An initial diagnosis of muscle spasm consistent with acute torticollis was rendered and the patient was prescribed symptomatic therapy with muscle relaxants and heat application. His pain generally resolved with only episodic recurrences. He returned to his PCP 2.5 months later for evaluation of a left upper arm painless lump that he had noticed 6–8 weeks prior to presentation. Of note, the bypass surgery graft was taken from this arm. He was referred to an orthopedic surgeon for further workup. A 5–8 cm mass was noted in the left posterior arm with mild tenderness over the left proximal humerus. There was no regional lymphadenopathy. The mass was nonmobile with no overlying erythema. Full range of motion was preserved. There were no neurological signs and the review of systems was negative. Plain films demonstrated subchondral sclerosis in the femoral head and a suspected permeative lesion in the proximal humerus. An ultrasound revealed an approximately 7 cm heterogeneous mass comprised of intramuscular soft tissue and fluid. An MRI (magnetic resonance imaging) showed a poorly defined 12.5 × 4.5 × 2.5 cm soft tissue mass infiltrating the lateral head of the triceps muscle (Figure 1). A potential marrow replacing lesions involving the humeral head and neck and proximal humeral shaft were also noted. A staging PET (positron emission tomography) revealed multiple FDG (fludeoxyglucose) avid intramuscular foci within the left posterior upper arm corresponding to the left triceps soft tissue mass noted on MRI as well as an additional FDG avid intramuscular nodule in the right suboccipital region posterior to the C1 vertebrae measuring 1.6 cm. There were also foci of mixed lytic/sclerotic lesions within the left proximal humeral diaphysis with increased FDG uptake. A concern for metastatic disease or lymphoma was raised and a CT (computed tomography) guided biopsy and fine needle aspiration (FNA) of the muscle and the bony lesions were performed (see Section 4). A diagnosis of primary muscle DLBCL was subsequently made. The patient underwent 5 cycles of R-CHOP (rituximab, cyclophosphamide, vincristine, doxorubicin, and prednisolone) therapy. A PET CT scan after the 2nd and 5th cycle revealed CR (complete remission). The patient refused the sixth and last cycle of R-CHOP. Radiation therapy was not included in the treatment plan since a CR was observed after the second cycle of R-CHOP. Currently the patient is feeling well except for some recurring neck pain for which he is receiving symptomatic therapy (heat application and ibuprofen).

## 3. Materials and Methods

### 3.1. Histology and Immunohistochemistry

Paraffin-embedded sections of the needle core biopsies were stained with hematoxylin and eosin (H&E). Immunohistochemical stains were performed on 4 *μ*m tissue sections using an Autostainer (Leica BOND platform, Buffalo Grove, IL). Sections were deparaffinized in xylene and graded alcohols. Detection of the antibodies was performed using a chromogenic substrate, diaminobenzene (Dako). The following antibodies were used: CD3 (RTU [ready to use], Leica), CD5 (RTU, Leica), CD10 (RTU, Leica), CD20 (L26) (RTU, Leica), CD30 (RTU, Leica), CD45 (1 : 200, Dako), CD79a (RTU, Leica), BCL6 (RTU, Leica), BCL2 (1 : 75, Dako), BCL1 (RTU, Fisher), c-MYC (1 : 100, Epitomics), PANCK (1 : 75, Biogenex), AE1/AE3 (1 : 400, Leica), CD138 (1 : 200, Dako), and Ki-67 (RTU, Dako).

### 3.2. Cytology

Diff-Quik and Papanicolaou smears were prepared following standard protocols. Briefly, for Diff-Quik stained sections direct smear preparations were allowed to fully air-dry. After this, slide was stained using HARLECO®Hemacolor Stain Set® (Fisher). First the slide was fully immersed in Hemacolor solution I, followed by staining in Hemacolor solution II for 30 sec, counterstaining in Hemacolor solution III for 30 sec, rinsing in distilled water, air drying, dipping in xylene, and mounting. For Papanicolaou staining, the slides were dipped in ethanol, followed by hematoxylin, 2% hydrochloric acid, ethanol, OG-6 solution, EA-65 solution, ethanol, and xylene.

### 3.3. Search for Internal Cases and Literature Search

To analyze clinicopathologic features of muscle lymphomas, we searched the electronic database of the Hospital of University of Pennsylvania between 2000 and 2015 for all cases of lymphoma involving skeletal muscle. A systematic literature search was conducted using the PubMed database with the following keywords: muscle lymphoma, skeletal muscle lymphoma, primary muscle lymphoma, and extranodal lymphoma/NHL. All published studies (case reports and case series) in the English language literature were reviewed with respect to presenting clinicopathologic features.

## 4. Results

### 4.1. Histology and Immunohistochemistry

**Figure 2:**
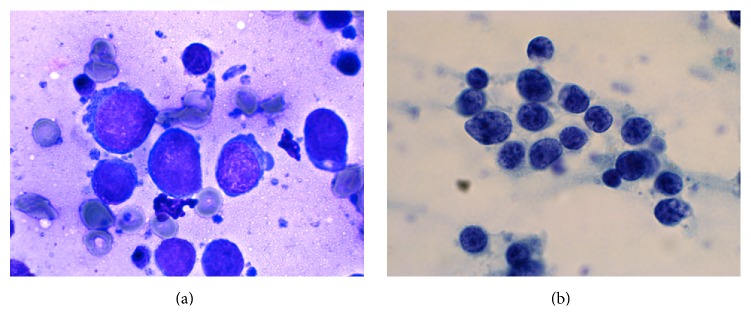
Diff-Quik (a) and Papanicolaou (b) stained smears from the FNA of the left lateral triceps mass show predominantly larger cells with occasional clustering.

**Figure 3:**
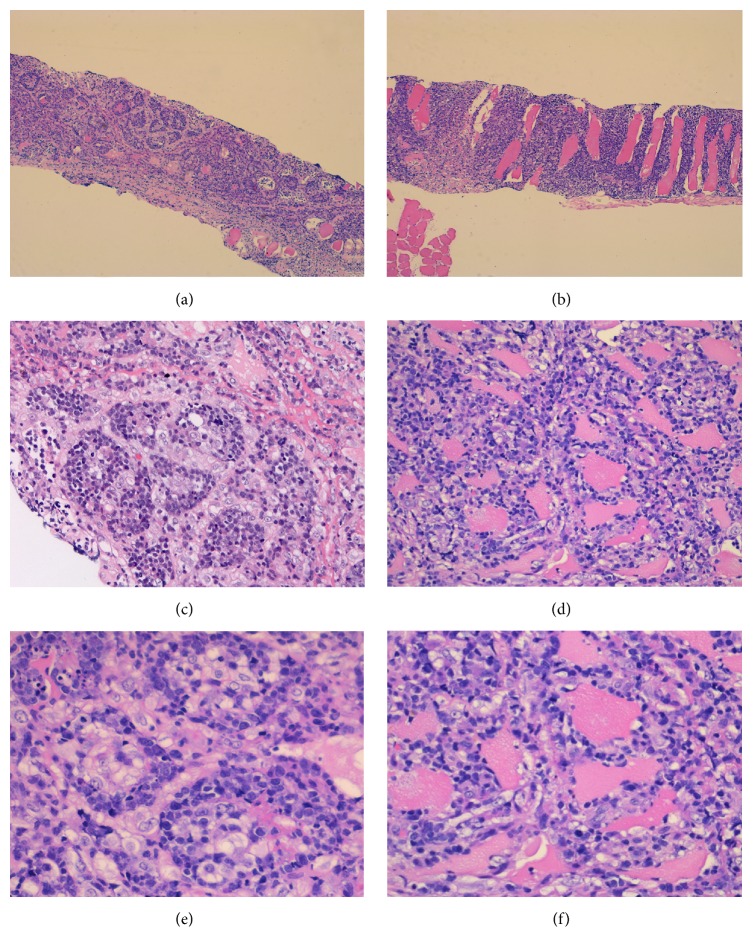
Histological findings of the muscle biopsy. Low power view (H&E stain, 5x) shows dense lymphoid infiltrate forming peculiar pseudoglandular structures (a) and sheets (b). Medium (H&E stain, 20x) and high power (H&E stain, 40x) stain view of the pseudoglandular structures (c and e) and areas densely infiltrating muscle tissue (d and f).

**Figure 4:**
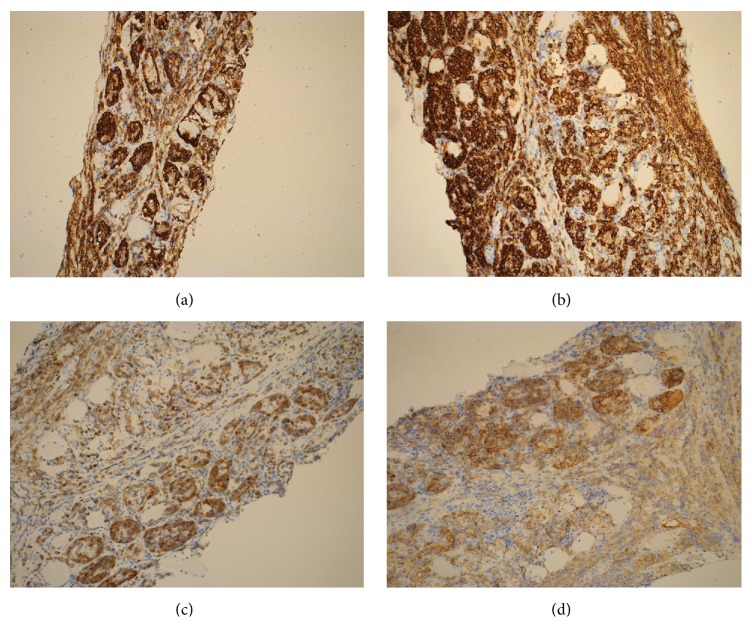
Immunohistochemical findings of the muscle biopsy. (a) Low power view (5x) reveals that CD45 stain highlights the pseudoglandular structures that are also immunoreactive for (b) CD20 (10x), (c) BCL2 (10x), and (d) CD10 (10x).

The FNA specimen from the left lateral triceps revealed predominantly large malignant cells with moderately open chromatin and occasional prominent nucleoli (Figures 2(a) and 2(b)). The material was insufficient for further workup. To better characterize the malignancy, a core biopsy of the lesion was performed. A portion of the specimen was submitted for flow cytometry. The H&E stained sections of the biopsy showed multiple cores of skeletal muscle diffusely involved by a dense lymphoid infiltrate forming pseudoglandular structures and sheets on low power examination (Figures 3(a) and 3(b)). On higher power examination, the cells were medium to large in size with irregular nuclear contours, vesicular chromatin, occasional prominent nucleoli, and moderate amounts of cytoplasm (Figures 3(c)–3(f)). Mitoses and apoptotic debris were abundant. Sections of bone did not reveal any evidence of the tumor (not shown). By immunohistochemistry, the infiltrating cells were immunoreactive for CD45 (LCA) (Figure 4(a)), CD20 (Figure 4(b)), CD79a, BCL2 (>50%) (Figure 4(c)), CD10 (dim) (Figure 4(d)), and BCL6 (subset) and were negative for cyclin D1, CD30, c-MYC, PANCK, AE1/AE3, and CD138. The Ki-67 proliferation index was approximately 50%. The material was insufficient for additional stains. Limited flow cytometric studies from the muscle tumor revealed an expansion of apparently large, surface immunoglobulin-negative, CD10 dim and CD19 positive, and CD5 negative cells.

The combined findings were consistent with a DLBCL, best classified as germinal center subtype by Hans classifier [10]. Due to absence of tumor at other locations this lesion was best classified as primary muscle DLBCL.

### 4.2. Review of Internal Cases and Literature Review

This unusual case prompted a review of all cases of lymphoma involving skeletal muscle diagnosed at our institution between 2000 and 2015 (Table 1). A total of 16 cases were identified and the majority (15/16) were NHL accounting for an incidence of skeletal muscle NHL at our institution of approximately 0.2% in the last 15 years. Of the 16 cases, 8 patients were male and 8 were female and the median age was 55 (range 36–85). The most common diagnosis was DLBCL (11/16), followed by marginal zone lymphoma (MZL) (2/16), anaplastic large cell lymphoma (ALCL) (1/16), follicular lymphoma (FL) (1/16), and Hodgkin lymphoma (HL) (1/16). The site of involvement was predominantly the lower extremities and pelvic muscles (6/16), followed by neck (5/16), head (4/16), and upper extremities (1/16). Eleven patients were treated with chemotherapy or chemoimmunotherapy most commonly with CHOP- (cyclophosphamide, adriamycin, vincristine, and prednisone-) like regimens with the addition of rituximab. Treatment outcomes were available for nine patients and seven patients were lost to follow-up. Whereas MZL, FL, ALCL, and HL showed a secondary involvement of the muscle, DLBCL showed a primary involvement in at least 3 cases based on the available information. Only 5 of the 16 cases were noted to be associated with lymphadenopathy.

A literature search was performed to evaluate clinicopathologic features of muscle cell lymphomas and the results are summarized in Table 2. Eighty-six patients were identified that fulfilled the search criteria, of whom 52 were men and 34 were women. The median age was 57 with the range of 5–90. As with our intrainstitutional cases, the most common lymphoma was DLBCL (61/86; 70.9%), followed by ALCL (6/86; 6.9%) and BL, FL, and PTCL (4/86; 4.7% each), indolent SBCL (3/86; 3.5%), NK/TCL (2/86; 2.3%), CLL (1/86; 1.2%), LBL (1/86; 1.2%), and MCL (1/86; 1.2%). The most common locations were the muscles of the lower extremities (36/86; 41.9%), followed by upper extremities (12/86; 13.9%), back (8/86; 9.3%), head/face (7/86; 8.1%), shoulder (7/86; 8.1%), pelvic (6/86; 6.9%), paraspinal (5/86; 5.8%), and rectal/anal muscles (5/86; 5.8%) and gluteal (5/86; 5.8%), chest (3/86; 3.5%), abdominal wall (2/86; 2.3%), and neck muscles (2/86; 2.3%). The majority of cases that included that information did not report associated lymphadenopathy. While many cases from the prerituximab era treated with a variety of chemotherapy regimens showed poor survival, the more recent cases treated with R-CHOP showed much better outcomes.

## 5. Discussion

We present a case of primary skeletal muscle DLBCL with an unusual pseudoglandular morphology. The morphologic, clinical, and genetic heterogeneity of DLBCL is well known but the features seen in this case are very uncommon [1, 11, 12]. Rare cases of pseudoglandular pattern have been described in ALCL [13] but to our knowledge this is first such case in DLBCL.

Extranodal manifestations of lymphoma, especially DLBCL, are well recognized and occur in up to 40% of DLBCL patients; however, primary skeletal muscle lymphoma is very rare accounting for less than 1% of cases [4–7, 14, 15]. These cases have been associated with a worse prognosis and a more advanced age. DLBCL is the most common histologic subtype of extranodal lymphoma involving skeletal muscle (Tables 1 and 2) [16, 17]. Infiltration of the muscle by lymphoma can mimic sarcoma, metastatic carcinoma melanoma, rhabdomyoblastoma, rhabdomyosarcoma, and osteosarcoma causing diagnostic difficulties [6]. It is critical to differentiate lymphoma from sarcoma and other entities to avoid unnecessary excisions. In contrast to sarcomas, lymphomas lack extracellular stroma and intercellular junctions. The neoplastic cells of lymphoma infiltrate more readily across anatomical fascial planes, frequently involving more than one muscle compartment [6, 16]. In addition lymphoma can exhibit confluent lymphadenopathy not seen in sarcoma [6]. The muscle involvement by lymphoma can be either primary or secondary (generally secondary to bone involvement). Frequently, cases of lymphoma involving skeletal muscle present with muscle pain, swelling, and a rapidly enlarging mass [5, 6]. The most commonly affected muscles are those of the extremities; however, other more unusual locations have been reported (Table 2) [5, 18]. Rare cases of DLBCL involving facial muscles and muscles of mastication can present with facial hemiplegia, paresthesia, and/or swelling [14, 19–21]. Muscle DLBCL can also be the cause of neurological complications due to nerve root entrapment and should be considered as an important cause of cranial peripheral nerve palsy or a piriformis syndrome [19, 22, 23]. Rarely, muscle lymphoma can present as an acute compartment syndrome [24]. It can also cause diffuse skin and soft tissue changes that may be suggestive of cellulitis [25] and has been reported to be a rare cause for a perianal abscess [26]. A potential contributing factor that may lead to the development of lymphoma in the muscle is a prior injury at that site. In fact, cases of primary muscle lymphoma following leg injury and needle injections and in rectum of homosexual men have been described [27–30]. Although NHL risk is increased in HIV-positive patients, primary muscle lymphomas in this patient population are rare with only few reported cases [14, 29–32].

A systematic study of 82 autopsy cases published in 1996 that investigated neoplastic infiltration in skeletal muscle in patients with lymphoma concluded that in contrary to scant literature reports on the subject skeletal muscle involvement in lymphoma is not such a rare phenomenon [33]. Muscle involvement was seen in 16% of lymphosarcoma (now designated NHL), 18% of reticulum cell sarcoma (now designated follicular dendritic cell tumor), and 6.8% of Hodgkin disease. The majority of the muscle groups affected were intercostal, psoas, diaphragm, and sternothyroideus muscles [33]. Another postmortem series of 194 patients who died of malignancy found skeletal muscle involvement in 20.6% of their lymphoma cases [34]. This raises a concern that muscle lymphoma may be more prevalent than currently appreciated.

Imaging studies are critical in the diagnostic workup of lymphoma in the muscle. MRI is the preferred technology to assess the extent of the mass within bone and surrounding tissues as it can offer higher soft tissue contrast and multiplanar imaging view [35–37]. Findings that are suggestive of primary muscular lymphoma include involvement of multiple muscular compartments and possible involvement of adjacent subcutaneous tissue and skin thickening. PET scan is mandatory for staging and evaluation of response and CT and scintigraphy play a complementary role and should only be used as initial examination and for staging purposes [28, 35, 36]. CT typically reveals nonspecific findings of a hypodense or isodense mass in the muscles that are not sufficient for accurate evaluation [38, 39]. Plain radiographs may be unremarkable or just show soft tissue swelling [36]. Sonography may show an ill-defined hypoechoic lesion, thickened fibroadipose septa, and swelling of soft tissue [40]. Although imaging studies are an important step in the workup of patients with muscle lymphoma, histological examination is necessary to confirm the diagnosis. Diagnosing of skeletal DLBCL on a small needle core biopsy especially in cases with unusual morphology may be very challenging and having a larger core biopsy with more material may be paramount for the diagnosis. Following the diagnosis of lymphoma, staging should be performed to select appropriate treatment.

The standard treatment for DLBCL is now combination chemotherapy and immunotherapy, known as R-CHOP (rituximab, doxorubicin, cyclophosphamide, vincristine, and prednisone) with or without radiation therapy but the presentation in unusual sites may require specific treatment approaches. Patients with extranodal NHL present more frequently with early stage disease compared with those with nodal NHL; overall survival in both groups largely depends on IPI (international prognostic index) and not on the site of origin of the malignancy [41]. Hence, although cases of primary skeletal DLBCL may be undertreated overall, if diagnosed early and correctly patients have a likelihood to achieve complete response upon treatment with R-CHOP [5, 42, 43].

In conclusion, primary skeletal muscle lymphoma is a rare entity and the majority of the cases belong to the group of DLBCL. MRI is the preferred technology in the diagnostic workup of potential skeletal muscle lymphoma. Due to a broad differential diagnosis of muscle lesions, a biopsy and histological examination are paramount for the diagnosis. The first-line treatment consists of R-CHOP; however modifications should be considered in more challenging cases.

## Figures and Tables

**Table 1:** Clinical characteristics, diagnosis, treatment, and follow-up in 16 patients with muscle lymphoma diagnosed at our institution between 2000 and 2015.

Location/muscle	Age	Gender	Diagnosis	Treatment	Follow-up
Piriformis muscle	50	M	DLBCL	R-CHOP + RT	Alive in CR at 6 years
Thigh muscle	55	M	ALCL, ALK−	CHOP/ONTAK	Lost to follow-up
Iliacus muscle	52	M	DLBCL	R-CHOP	Died at 3 months
Psoas muscle	60	M	DLBCL	R-CE	Alive with PD at 28 months
Triceps muscle	79	M	DLBCL	R-CHOP	Alive in CR at 6 months
Psoas muscle	70	F	DLBCL	NR	NR
Sternothyroid muscle	57	F	DLBCL	R-CHOP + RT	Alive in CR at 14 years
Temporalis muscle	45	M	MZL	FCR	Alive in CR at 10 years
Cervical muscle	78	M	DLBCL	R-CHOP	Alive in CR at 10 years
Iliacus muscle	85	F	DLBCL	R-CHOP + RT	Died at 24 months
Buccal muscle	36	M	DLBCL	NR	Lost to follow-up
Right buccal	55	F	DLBCL	NR	Lost to follow-up
Buccal muscle	50	F	FL, low-grade	R-CHOP	Lost to follow-up
Strap muscle	42	F	HL	ABVD	Alive in CR at 10 years
Neck muscles	54	F	DLBCL	NR	Lost to follow-up
Neck muscles	79	F	MZL	NR	Lost to follow-up

ABVD = adriamycin, bleomycin, vinblastine, and dacarbazine; ALCL = anaplastic large T-cell lymphoma; ALK = anaplastic lymphoma kinase; CHOP = cyclophosphamide, adriamycin, vincristine, and prednisone; CR = complete response; DLBCL = diffuse large B-cell lymphoma; F = female; FCR = fludarabine, cyclophosphamide, and rituximab; FL = follicular lymphoma; HL = Hodgkin lymphoma; M = male; MZL = marginal zone lymphoma; NR = not reported; ONTAK = denileukin diftitox; PD = progressive disease; R-CHOP = rituximab, cyclophosphamide, doxorubicin, vincristine, and prednisone; R-CE: rituxan-cytoxan-etoposide; RT = radiation therapy.

**Table 2:** Clinicopathologic characteristics of 86 patients from the literature with reported muscle lymphoma.

*Patients*, *n*	86
*Sex*, male/female	52/34
*Age* median (range)	57 (5–90)
<18 years	5

Histology	Patients, *n* (%)

DLBCL	61 (70.9)
ALCL^*∗*^	6 (6.9)
BL	4 (4.7)
FL	4 (4.7)
PTCL	4 (4.7)
Indolent SBCL, type not specified	3 (3.5)
NK/TCL	2 (2.3)
CLL	1 (1.2)
LBL	1 (1.2)
MCL	1 (1.2)

Location	Patients, *n* (%)

Lower extremities	36 (41.9)
Upper extremities	12 (13.9)
Back	8 (9.3)
Head/face	7 (8.1)
Shoulder	7 (8.1)
Pelvic	6 (6.9)
Paraspinal	5 (5.8)
Rectal/anal	5 (5.8)
Gluteal	5 (5.8)
Chest	3 (3.5)
Abdominal wall	2 (2.3)
Neck	2 (2.3)

ALCL = anaplastic large T-cell lymphoma; BL = Burkitt lymphoma; CLL = chronic lymphocytic leukemia; DLBCL = diffuse large B-cell lymphoma; FL = follicular lymphoma; LBL = lymphoblastic lymphoma; MCL = mantle cell lymphoma; NK/TCL = NK/T-cell lymphoma; PTCL = peripheral T-cell lymphoma; SBCL = small B-cell lymphoma.

^*∗*^3 cases with ALK+/2 cases ALK−/1 case ALK not reported.
